# Emergency Department Visits, Hospital Admissions, and Wait Times for Patients With Urologic Conditions

**DOI:** 10.1001/jamanetworkopen.2025.60058

**Published:** 2026-03-09

**Authors:** Rano Matta, Jordyn Shaw, Hodan Mohamud, Samantha Morais, Refik Saskin, Amanda Hird, Sarah Neu, Sender Herschorn, Robert K. Nam

**Affiliations:** 1 Division of Urology, University of Toronto, Department of Surgery, Toronto, Ontario, Canada; 2 Division of Urology, Sunnybrook Health Sciences Center, Toronto, Ontario, Canada; 3 Temerty Faculty of Medicine, University of Toronto, Toronto, Ontario, Canada; 4 ICES, Toronto, Ontario, Canada; 5 Institute for Health Policy, Management & Evaluation, University of Toronto, Toronto, Ontario, Canada

## Abstract

**Importance:**

Increased use of the emergency department (ED) creates strain on the single-payer public health care system in Canada.

**Objective:**

To evaluate trends in ED visits and hospital admission rates for urologic conditions in Ontario, Canada, and to determine trends and associated factors for wait times to see a urologist among patients presenting to the ED.

**Design, Setting, and Participants:**

This population-based, retrospective cohort study used health administrative data from all EDs and acute care hospitals in Ontario for all adult patients presenting to the ED with a primary urological diagnosis between January 1, 2007, and December 31, 2022. Analyses were performed from January 2023 to April 2024.

**Main Outcomes and Measures:**

The main outcome was the rate of ED visits with new urologic diagnoses over time. Crude, age-standardized, and sex-standardized annual rates were estimated for index ED visits and hospital admissions. Wait times to see a urologist after ED visit were also determined.

**Results:**

A total of 2 192 213 unique visits to the ED with a main diagnosis of a urologic disorder were identified (mean [SD] patient age, 52.1 [21.2] years; 66.5% female). Of these, 1 732 356 visits represented a new urologic diagnosis (no visits in the prior 2 years). Age- and sex-standardized ED visit rates rose annually from 2007 (0.91 visits per 100 people) to 2015 (1.0 visits per 100 people) (rate ratio [RR], 1.10; 95% CI, 1.09-1.11), then decreased until 2020 (RR, 0.88; 95% CI, 0.87-0.88), before rising again until 2022 (RR, 0.91; 95% CI, 0.90-0.91). Among people seen in the ED with a urologic diagnosis, 10.0% were admitted to the hospital during the study period. The crude rate of admission rose annually (RR, 1.04; 95% CI, 1.03-1.05; *P* < .001), from 0.07 (95% CI, 0.07-0.08) admissions per 100 people in 2007 to 0.09 (95% CI, 0.09-0.09) in 2022. Patients with greater continuity of outpatient care had lower odds of being admitted. The mean (SD) wait time to see a urologist after an ED visit increased from 62.5 (80.3) days in 2007 to 84.8 (89.3) days in 2014. It subsequently decreased annually until 2022, to 71.1 (70.6) days. Increased comorbidity was associated with lower risk of outpatient urology visit.

**Conclusions and Relevance:**

In this cohort study, the annual ED visit rates for new urologic diagnoses, hospital admissions, and wait times to see urologists significantly increased over the study period. These trends were associated with decreasing continuity of care. This increasing burden of acute urologic disease necessitates investment in health care.

## Key Points

**Question:**

What is the trend over time in emergency department (ED) visits, hospital admission rates, and time to see an outpatient specialist for urologic conditions in Ontario, Canada?

**Findings:**

In this cohort study that included 2.19 million individual ED visits from 2007 to 2022, annual rates of ED visits for new urologic diagnoses, hospital admissions, and wait times to see urologists significantly increased during the study period.

**Meaning:**

These findings suggest a rising burden of acute urologic disease, which necessitates investment in health care resources and efficient resource allocation.

## Teaser

This cohort study examines trends in emergency department (ED) visits, hospital admission rates, and wait times to see a urologist among patients presenting to the ED with urologic conditions.

## Introduction

Challenges in timely access to primary and specialist care are pain points among publicly funded health care systems. In Canadian health care, which is a publicly funded, single-payer system, timely access has become a pressing issue for many nonurgent health services. Among the Organization for Economic Co-operation and Development nations from the 2016 Commonwealth Fund International Health Policy Surveys, Canada has consistently ranked as having the largest proportion of people who were unable to get same-day responses from their primary care office (33%) and people waiting 1 month or longer for a specialist visit (61%).^1^ Recent audit of charts of primary care clinics in Canada found that the median national wait time in Canada for seeing a specialist after referral was 78 days.^2^ The lack of primary care has been associated with increased in-hospital mortality and mortality within 1 year of hospital admission.^3^ In addition, limited primary care and limited continuity of care are associated with increased use of the emergency department (ED).^4^ ED visits have risen in Ontario, Canada’s most populous province, and outpaced population growth for the last 7 years, with a 13.4% increase in the annual number of ED visits compared to a 6.2% increase in the province’s population.^5^

The burden of acute urological presentations to the ED in Ontario has followed a similar trajectory.^6,7^ With a growing and aging population in the province, as in many Western nations, it is necessary to understand the impact of primary and specialist care access on emergency and nonemergency urological presentations in order to appropriately allocate resources and minimize morbidity among people seeking care.

Therefore, within the province of Ontario, Canada, we sought to evaluate temporal trends in ED visits for new urologic conditions, rates of hospital admission, and subsequent wait time to see a urologist. We hypothesized that ED visits for new urologic conditions were increasing over time. We also aimed to determine factors associated with annual ED visit rate, hospital admissions, and wait times, including age, comorbidity, geographic location, socioeconomic status, and continuity of care. We hypothesize that when people do not have regular continuity of care, they are increasingly seeking care for new urologic problems in the ED, more likely to be admitted to hospital, and are waiting longer to see a urologist in an outpatient setting.

## Methods

### Data Sources and Setting

We conducted a retrospective cohort study of adults (≥18 years old) presenting to the ED with a primary urological diagnosis (listed in eTable 1 in Supplement 1) between January 1, 2007, and December 31, 2022, in Ontario, Canada using linked health administrative databases. In Ontario, all necessary health care services and physician services information are recorded and held at the ICES.^8^ Each of the data sources used has been validated previously (eMethods in Supplement 1). The Sunnybrook Health Sciences Center Research Ethics Board approved the study protocol. We followed the Strengthening the Reporting of Observational Studies in Epidemiology (STROBE) reporting guideline.

### Study Participants

We identified all people aged 18 years or older who presented to the ED from January 1, 2007, to December 31, 2022. We selected a cohort of people presenting with a new urological diagnosis (eTable 1 in Supplement 1), taking the first episode in each calendar year, and excluding individuals with a urologic-related visit to the ED or to a urologist (inpatient or outpatient) in the previous 2 years. We also excluded people older than 105 years at presentation; people with invalid birth dates, death dates, or sex; non–Ontario residents; people ineligible for health care in the province of Ontario within 6 months prior to their ED visit; people who were pregnant; and those admitted with trauma or injury due to external causes. Cohort selection is shown in eTables 2 and 3 in Supplement 1.

### Covariates

We collected patient baseline characteristics that may confound the association between the year of index ED visit and the hospital admission and wait time to see a urologist. Patient age, sex, Charlson Comorbidity Index (CCI) score (a measure of comorbidities associated with mortality, with scores from 0 to ≥3, ≥3 being the most comorbid), rurality, and income quintile were measured. CCI was measured with a 24-month lookback period prior to the ED index visit. We measured continuity of care using the Bice-Boxerman Continuity of Care Index (COCI), measured with a 12-month lookback period prior to the ED index visit.^9^ The COCI spans from 0 to 1, with 0 denoting that all of a person’s visits are with a different, unreferred physician (for example, if a person saw only different primary care physicians in urgent care) and 1 denoting perfect continuity, where all physician visits were with or referred by the same physician.

### Outcomes

The primary outcome was hospital admission following ED visit. Secondary outcomes were age- and sex-standardized rates of index ED visits with a primary urologic diagnosis and wait times to see a urologist.

### Analysis

We calculated crude, age-standardized, and sex-standardized rates of all index ED visits and index ED visits with a urologic diagnosis. Annual incidence rates of hospital admissions were also determined for all index ED visits and index ED visits with a urologic diagnosis. We calculated the wait time to see a urologist based on the time from ED visit to the first subsequent appointment with any urologist. To determine factors associated with hospitalization among people visiting the ED with a urologic diagnosis, we generated multivariable models (by year and overall), adjusting for the aforementioned covariates. We also generated multivariable hazard models to determine factors associated with wait times for visit to outpatient urology specialist (within 1 year).

### Statistical Analysis

Age- and sex-standardized rates were calculated by year using direct standardization. The denominator was the total number of Ontario residents eligible for the Ontario Health Insurance Plan (OHIP) each year, and the standard population was the 2014 Ontario population eligible for OHIP with 5-year age groups (18-19, 20-24, ..., 85-89, and ≥90 years). Rate ratios and 95% CIs were estimated using the EFFECT option (PROC STDRATE in SAS), which computes the rate effect between the study populations (years, with 2007 as the reference) with the default rate ratio statistics. Annual incidence rate ratios were estimated using Poisson regression with a deviance scale adjustment to correct for overdispersion and a logarithm of the population offset (provides the denominator of the outcome). The same was done for estimating hospital admission rates following an index ED visit.

To determine factors associated with hospitalization among people visiting the ED with a urologic diagnosis, we generated multivariable logistic regression models (by year and overall). We generated hazard models (by year and overall) to determine factors associated with wait times for a visit to an outpatient urology specialist (within 1 year). The proportional hazards assumption was evaluated using Shoenfeld residuals. Statistical analyses were performed using SAS Enterprise Guide, version 8.3 (SAS Institute). All analyses were performed between January 2023 and April 2024.

## Results

**Figure 1.**
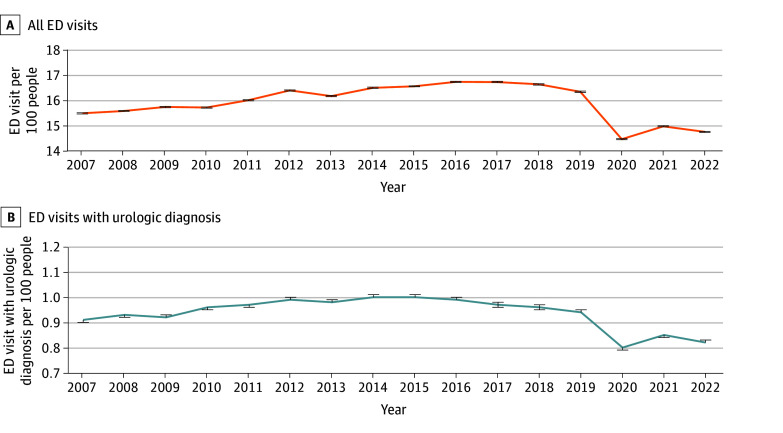
Line Graphs of Rates of Emergency Department (ED) Visits per 100 People From 2007 to 2022 in Ontario, Canada Rates for all ED visits (n = 29 461 281) (A) and for ED visits with urologic diagnosis (n = 2 192 213) (B). Error bars represent 95% CIs.

We identified 2 192 213 unique ED visits with a main diagnosis of a urologic disorder between 2007 and 2022. Patients’ mean (SD) age was 52.1 (21.2) years, 66.5% were female, and 33.5% were male (eTable 3 in Supplement 1 for cohort selection). When we excluded those with a urologic inpatient or outpatient visit in the previous 2 years, we identified 1 732 356 ED visits involving new urologic diagnoses. The baseline characteristics of patients seen in the ED are summarized in eTable 4 in Supplement 1. The top 5 most common diagnoses for patients seen in the ED and discharged are listed in Table 1, with urinary tract infection (UTI) being the most common, present in 1 017 631 ED visits (65.7%). Between 2007 and 2019, the rate of ED visits for urological diagnoses increased annually (rate ratio [RR], 1.01; 95% CI, 1.00-1.02) from 0.91 (95% CI, 0.90-0.91) visits per 100 people in 2007 to 1.00 (95% CI, 1.00-1.10) visits in 2015 (RR for 2007 to 2015, 1.10; 95% CI, 1.09-1.11), then decreasing annually until 2020 (RR for 2007 to 2020, 0.88; 95% CI, 0.87-0.88), with the largest drop between 2019 (RR, 0.94; 95% CI, 0.94-0.95) and 2020 (RR, 0.80; 95% CI, 0.79-0.80) (Figure 1). There was then a rise in the annual ED visit rate from 2020 to 2022, with an RR of 0.82 (95% CI, 0.82-0.83) in 2022 (RR for 2007 to 2022, 0.91; 95% CI, 0.90-0.91) (Figure 1; eTable 5 in Supplement 1). ED visit rates for urologic disorders were higher over the whole study period (2007-2022) for females (1.22 [95% CI, 1.22-1.22] visits per 100) than for males (0.65 [95% CI, 0.65-0.66] visits per 100).

**Table 1.** Top 5 Urologic Diagnoses for Patients Presenting to the Emergency Department (ED) for New Urological Diagnoses by Admission vs Discharge

Diagnosis	Visits, No. (%)
Discharged from ED (n = 1 548 641)	
Urinary tract infection	1 017 631 (65.7)
Kidney colic and stones	292 917 (18.9)
Disorders of male genital organs	136 438 (8.8)
Obstructive and reflux uropathy	53 400 (3.5)
Prostatitis	12 739 (0.8)
Admitted to hospital (n = 174 019)	
Urinary tract infection	90 363 (51.9)
Acute kidney failure, unspecified	68 410 (39.3)
Kidney colic and stones	7515 (4.3)
Obstructive and reflux uropathy	4564 (2.6)
Disorders of male genital organs	1864 (1.1)

**Figure 2.**
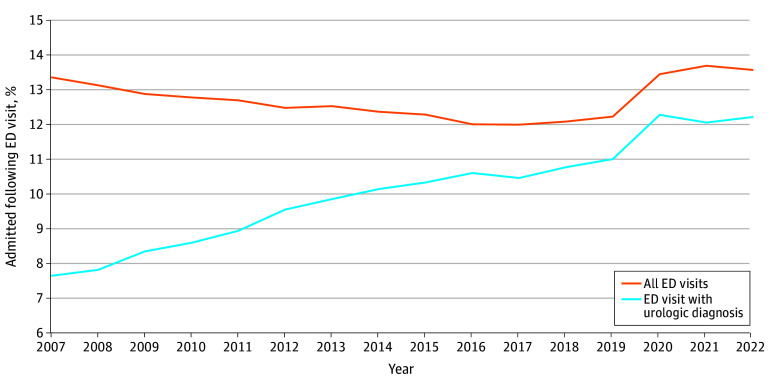
Line Graph of Proportion of Hospital Admission Following an Emergency Department (ED) Visit From 2007 to 2022 in Ontario, Canada

A total of 174 017 unique ED visits with a primary diagnosis of a urologic disorder resulted in admission to the hospital (10.0%). The most common diagnoses for patients seen in the ED and admitted to the hospital are listed in Table 1, with UTI being the most common, present in 90 363 hospital admissions (51.9%). The proportion of people admitted to the hospital (of all visits with primary urologic diagnosis) increased steadily from 2007 (7.7%) to 2022 (12.2%) (Figure 2). Between 2007 and 2022, the rate of admission increased annually (RR, 1.04; 95% CI, 1.03-1.05; *P* < .001) from 0.07 (95% CI, 0.07-0.08) admissions per 100 people in 2007 to 0.09 (95% CI, 0.09-0.09) in 2022. During this period, the rate of admissions for all ED visits (all diagnoses) increased annually (RR, 1.01; 95% CI, 1.01-1.02; *P* < .001), with the proportion of people admitted to hospital increasing from 13.4% in 2007 to 13.6% in 2022.

**Figure 3.**
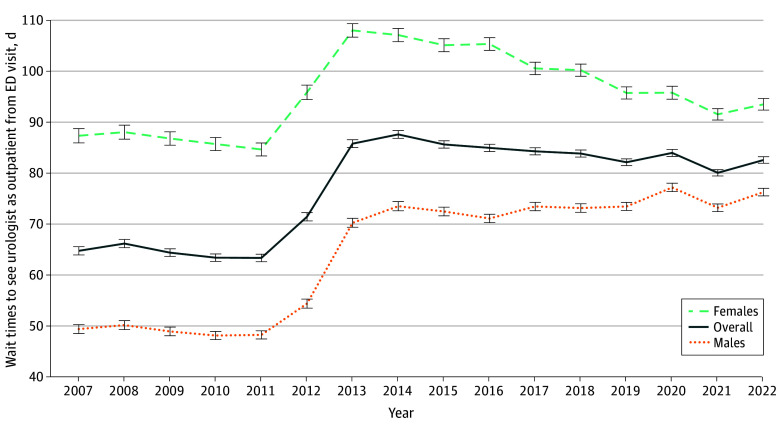
Line Graph of Median Time to See an Outpatient Urologist Following an Emergency Department (ED) Visit From 2007 to 2022 in Ontario, Canada The graph represents 239 416 visits to an outpatient urology specialist during the study period. Error bars represent the standard error of the mean.

A total of 239 416 visits to an outpatient urology specialist were captured following an ED visit for a new urologic diagnosis (14.1% of all ED visits). The proportion of ED visits resulting in an outpatient visit increased from 10.97% in 2007 to 15.30% in 2022 (RR, 1.03; 95% CI, 1.03-1.04; *P* < .001). The mean (SD) wait time to see a urologist following an ED visit as an outpatient was 78.3 (85.1) days, increasing from 62.5 (80.3) days in 2007 to 84.8 (89.3) days in 2014 (Figure 3). It subsequently decreased annually until 2022, to a mean (SD) of 71.1 (70.6) days (Figure 3). The mean (SD) wait time from 2007 to 2022 to see a urologist for female patients was 96.3 (93.4) days; the mean (SD) wait time from male patients was 66.5 (76.8) days (Figure 3).

With each increase in year of age, individuals were more likely to be admitted to the hospital (odds ratio [OR], 1.06; 95% CI, 1.06-1.06). Females had lower odds of hospital admission compared with males (OR, 0.68; 95% CI, 0.67-0.69). Individuals with higher comorbidity were more likely to be admitted to hospital than those with low comorbidity. Individuals with a higher continuity of care in the community, based on the COCI, were less likely (OR, 0.83; 95% CI, 0.82-0.84) to be admitted to the hospital (Table 2).

**Table 2.** Association Between Patient Characteristics and Hospital Admission and Wait Time to See Urologist as an Outpatient Following Emergency Department (ED) Visits for New Urological Diagnoses^a^

Characteristic	Hospital admission, OR (95% CI)	Wait time to see urologist, HR (95% CI)
Age	1.06 (1.06-1.06)	1.01 (1.01-1.01)
Sex		
Male	1 [Reference]	1 [Reference]
Female	0.68 (0.67-0.69)	0.28 (0.28-0.29)
Income quintile		
5 (Highest)	1 [Reference]	1 [Reference]
4	1.24 (1.22-1.27)	0.99 (0.98-1.00)
3	1.15 (1.12-1.17)	0.95 (0.93-0.96)
2	1.06 (1.04-1.08)	0.93 (0.92-0.95)
1 (Lowest)	1.00 (0.98-1.02)	0.88 (0.86-0.89)
Charlson Comorbidity Index		
0	1 [Reference]	1 [Reference]
1	1.77 (1.74-1.80)	0.90 (0.89-0.92)
2	4.99 (4.89-5.08)	0.75 (0.73-0.76)
≥3	6.87 (6.75-6.99)	0.57 (0.55-0.58)
Continuity of care^d^		
Very low	1 [Reference]	1 [Reference]
None	1.38 (1.31-1.44)	0.88 (0.85-0.91)
Low	0.80 (0.78-0.81)	1.20 (1.19-1.22)
Medium	0.81 (0.79-0.83)	1.31 (1.29-1.34)
High	0.83 (0.82-0.84)	1.15 (1.14-1.16)

^a^ Patient data from first ED visit in study period (N = 1 548 641). The model was adjusted for age, sex, income, Charlson Comorbidity Index score, and Bice-Boxerman Continuity of Care Index.

^d^ Bice-Boxerman Continuity of Care Index.

With each increase in year of age, individuals were less likely to wait to see an outpatient urologist (hazard ratio [HR], 1.01; 95% CI, 1.01-1.01). Females had a significantly longer wait time to see a urologist than males (HR, 0.28; 95% CI, 0.28-0.29). Individuals in the lowest income quintile had the highest risk of waiting (HR, 0.88; 95% CI 0.86-0.89) compared with the highest income quintile. Individuals with higher comorbidity were more likely to have longer wait times to see an outpatient urologist; patients with a CCI score of ≥3 had an increased risk of longer wait (HR, 0.57; 95% CI, 0.55-0.58) compared with those with a score of 0. Individuals with a high COCI had earlier follow-up with outpatient urology than those with a very low COCI (HR, 1.15; 95%CI 1.14-1.16) (Table 2; eTable 6 in Supplement 1).

## Discussion

This cohort study evaluates trends in ED visits for new urologic conditions, hospital admission rates, and wait times to see a urologist in Ontario, Canada, from 2007 to 2022. To our knowledge, this is the first population-based study evaluating ED use for urological disorders and its association with access to primary and specialist care. We identified that the annual rate of people visiting the ED for a new urologic diagnosis has significantly increased over time, with rising rates of hospital admission and increased wait times to see an outpatient urologist.

Over the study period, trends in rates of ED visits for new urologic conditions reflected trends observed for all ED visits. We observed an approximately 1% year-over-year increase in the rate of both total and urologic ED visits per 100 people from 2007 until 2019, followed by a decrease from 2019 to 2020. This decrease during the COVID-19 pandemic is consistent with similar data for all ED visits reported in Canada^10^ and other countries worldwide.^11,12,13,14,15,16^ From 2020 to 2022, we observed a small annual rise in ED visit rates, albeit at a slower pace compared to prepandemic rates. Overall, the trends are consistent with data reported by the Canadian Institute for Health Information (CIHI) National Ambulatory Care Reporting System (NACRS) during the same period.^14^

Although we observed similar trends between urologic-related and total ED visit rates, trends in hospital admission rates from these ED visits diverged between 2007 and 2022. The proportion of all ED visits resulting in patients admitted to the hospital increased slightly from 13.4% to 13.6% over the same period. By comparison, the proportion of people admitted to the hospital with a primary urologic diagnosis increased steadily year over year from 7.7% in 2007 to 12.2% in 2022 and appears to converge on the overall admission rate in Ontario. The average proportion of people admitted for urologic diagnosis during our study period was 10%. This is in keeping with previous studies from the US, which report admission rates of 8% for kidney colic^17^ and 12% for people with upper tract stones.^18^ Similarly, admissions for UTI in Japan between 2011 and 2015 were estimated at 0.07 per 100 males to 0.12 per 100 females,^19^ in line with our data showing an admission rate of 0.07 to 0.09 per 100 people in 2007 to 2022, respectively.

We observed female sex to be independently associated with a lower likelihood of hospitalization following ED visit with urologic diagnosis compared with male patients. We surmise that this is due to a larger proportion of lower-acuity presentations to ED with treatment in an outpatient setting for UTI in female patients compared with male. UTI incidence is known to be much higher in female patients compared with male patients,^20,21^ and outpatient treatment is more common and has overtaken inpatient treatment of UTI.^22^

We found increased age, increased comorbidity, and lower income to be associated with a higher risk of admission. This finding is consistent with a consensus in the literature that these factors are associated with worse health care outcomes and an increased likelihood of hospitalization.^19,23,24,25,26^ In the context of urological diagnoses, age is well known to be associated with higher rates of admissions for both UTI^27,28,29^ and acute kidney failure.^30,31^

We observed a higher continuity of care to be associated with a decreased risk of admission following ED visits for urologic diagnosis. This finding agrees with other studies in Canada^32^ and worldwide^33,34^ that show high continuity of care to be associated with an overall lower likelihood of hospitalization, especially among older patients. We found that patients with too few outpatient visits to calculate a continuity of care index, categorized as none, had a higher likelihood of hospital admission for urological conditions. Our results, together with similar findings for general ED visit rates and admission rates reported worldwide,^4,35,36^ highlight the need for increased access to health care in the community and continuity of care to improve patient outcomes and reduce the burden on hospital systems.^37^

Another measure of ambulatory patient care is timely access to specialist services. A Canadian study by Prudhomme et al^38^ estimated 12% of patients discharged from the ED are referred to an outpatient specialist. This is comparable to our results, showing 14% of patients were subsequently seen in the outpatient urology setting. The mean wait time to see a urologist as an outpatient from ED was 78.3 days. This is consistent with previously reported wait times to see a urologist following referral from primary care clinics in Canada at a median of 75.5 days reported by Liddy et al^2^ and Neimanis et al^39^ reporting a mean wait time of 64 days in Ontario. Referrals to outpatient specialists have been increasing year over year.^40^ An increased volume of referrals from primary care and other specialist physicians, coupled with resource constraints on urologist hiring,^41^ likely contribute to the increasing wait times observed in our study.

The patient factors we observed to be associated with longer wait times were female sex, lower income, higher comorbidity index, and no continuity of care. The association of wait times with patient sex is inconsistent in the literature, and it remains unclear if this is specific to urology. Some previous studies of wait time in Ontario did not show an independent association of patient sex with wait time to outpatient specialists in general^42^ or urologists specifically,^43^ while others showed females had longer wait times.^44^ Johnson et al^45^ demonstrated a disparity in referral rates for hematuria in men compared with women, with women being referred to a urologist less often than men for the same symptoms. Further investigation into potential discrepancies between wait times for males and females to see urologists in an outpatient setting are warranted.

In this study, we noted that lower income was independently associated with an increased likelihood of admission following ED visits for urologic diagnosis and is also associated with increased odds of longer wait times to see a urologist as an outpatient. The association of lower socioeconomic status (SES) factors, including income, with longer wait times for specialty procedures and appointments is well established in Canada and worldwide.^46,47,48,49,50^ This similarity in patient factors associated with both increased admission and longer outpatient wait time warrants further investigation.

### Strengths and Limitations

A major strength of our study is the large dataset spanning 15 years of all electronic medical records in Ontario. This allows for a comprehensive longitudinal perspective on ED visit rates, hospital admission rates for urological diagnoses, and wait times for outpatient urologist appointments. This is especially valuable because many studies of wait times rely on patient and/or clinician surveys, which have low response rates (often <30%) and are subject to recall and other biases.^2,39,51^ A limitation is that our study only includes data from Ontario. Although this is the largest and most populated province in Canada (14.2 million people in 2021),^52^ our findings are not representative of all health systems in Canada and may not be generalizable to other countries with a public funded health care system. Additionally, our study does not account for frequent users of the ED, who represent a small proportion of the overall ED patient population (approximately 5%) but approximately 25% of all ED visits^53^ and have similar characteristics worldwide.^54^ The omittance of this patient population, which is a large contributor to ED overcrowding and high health care costs, represents a source of bias. Future investigations including this patient population may provide a more representative view of the patient characteristics and trends in the use of urology services in Ontario.

## Conclusions

In this population-based retrospective cohort study of people presenting to Canadian emergency departments, the annual rate of patients visiting the ED with primary urologic diagnoses significantly increased between 2007 and 2022, accompanied by rising hospital admission rates. Wait times to see a urologist after ED visits also increased. Limited access to care in an ambulatory setting was associated with an increased rate of hospital admission and longer wait times to see a urologist. This work emphasizes the importance of enhancing primary care access and continuity to mitigate hospitalizations and reduce wait times for people with urologic conditions.

## Notes

Supplement 1. **eTable 1.** Urologic diagnoses captured using ICD-10 codes
eMethods.
**eTable 2.** Cohort Selection for all emergency department visits **eTable 3.** Cohort Selection for all emergency department visits with a primary urologic diagnosis **eTable 4.** Baseline characteristics of patients seen in the emergency department with a primary urological diagnosis for a new urological condition **eTable 5.** Age- and sex- standardized rates of all unique ED visits and a first ED visit with a primary urological diagnosis for new urologic conditions between Jan 1 2007 and Dec 31 2022 by year **eTable 6.** Association between patient characteristics and hospital admission and wait time to see urologist as an outpatient following emergency department (ED) visits for new urological diagnoses, crude model

Supplement 2.
Data Sharing Statement
