# Supplementary material for: Emergency Department Visits, Hospital Admissions, and Wait Times for Patients With Urologic Conditions
Source: JAMA Netw Open. 2026 Mar 9;9(3):e2560058. doi: 10.1001/jamanetworkopen.2025.60058 (PMC12973099; doi:10.1001/jamanetworkopen.2025.60058)
Supplement: Supplement 1. — eTable 1. Urologic diagnoses captured using ICD-10 codes eMethods. eTable 2. Cohort Selection for all emergency department visits eTable 3. Cohort Selection for all emergency department visits with a primary urologic diagnosis eTable 4. Baseline characteristics of patients seen in the emergency department with a primary urological diagnosis for a new urological condition eTable 5. Age- and sex- standardized rates of all unique ED visits and a first ED visit with a primary urological diagnosis for new urologic conditions between Jan 1 2007 and Dec 31 2022 by year eTable 6. Association between patient characteristics and hospital admission and wait time to see urologist as an outpatient following emergency department (ED) visits for new urological diagnoses, crude model [file jamanetwopen-e2560058-s001.pdf]

Supplemental Online Content

Matta R, Shaw J, Mohamud H, et al. Emergency department visits, hospital admissions, and wait times for patients with urologic conditions. *JAMA Netw Open*. 2026;9(2):e2560058. doi:10.1001/jamanetworkopen.2025.60058

eTable 1. Urologic diagnoses captured using ICD-10 codes

eMethods.

eTable 2. Cohort Selection for all emergency department visits

eTable 3. Cohort Selection for all emergency department visits with a primary urologic diagnosis

eTable 4. Baseline characteristics of patients seen in the emergency department with a primary urological diagnosis for a new urological condition

eTable 5. Age- and sex- standardized rates of all unique ED visits and a first ED visit with a primary urological diagnosis for new urologic conditions between Jan 1 2007 and Dec 31 2022 by year

eTable 6. Association between patient characteristics and hospital admission and wait time to see urologist as an outpatient following emergency department (ED) visits for new urological diagnoses, crude model

This supplemental material has been provided by the authors to give readers additional information about their work.

© 2026 Matta R et al. JAMA Network Open.

eTable 1. Urologic diagnoses captured using ICD-10 codes

Full ICD-10 code	Full digit ICD-10 code description
**Bladder Dysfunction**	
N304	Irradiation cystitis
N310	Uninhibited neuropathic bladder, not elsewhere classified
N311	Reflex neuropathic bladder, not elsewhere classified
N312	Flaccid neuropathic bladder, not elsewhere classified
N323	Diverticulum of bladder
N328	Other specified disorders of bladder
N329	Bladder disorder, unspecified
N393	Stress incontinence
N3930	Mixed incontinence
N3939	Stress incontinence
N394	Other specified urinary incontinence
N398	Other specified disorders of urinary system
N399	Disorder of urinary system, unspecified
**Female Genital Disorders**	
N810	Female urethrocele
N811	Cystocele
N818	Other female genital prolapse
**Fistula**	
N321	Vesicointestinal fistula
N322	Vesical fistula, not elsewhere classified
N360	Urethral fistula
N820	Vesicovaginal fistula
N821	Other female urinary-genital tract fistulae
N825	Female genital tract-skin fistulae
**Hemorrhage**	
N028	Recurrent and persistent haematuria, other
N029	Recurrent and persistent haematuria, unspecified
N5010	Hematospermia
**Infection**	
N110	Nonobstructive reflux-associated chronic pyelonephritis
N111	Chronic obstructive pyelonephritis
N136	Pyonephrosis
N151	Renal and perinephric abscess
N300	Acute cystitis
N301	Interstitial cystitis (chronic)
N302	Other chronic cystitis
N303	Trigonitis
N308	Other cystitis
N309	Cystitis, unspecified
N340	Urethral abscess
N341	Nonspecific urethritis
N342	Other urethritis
N343	Urethral syndrome, unspecified
N370	Urethritis in diseases classified elsewhere
N390	Urinary tract infection, site not specified
**Nephrolithiasis**	
N132	Hydronephrosis with renal and ureteral calculous obstruction
N200	Calculus of kidney
N201	Calculus of ureter

© 2026 Matta R et al. JAMA Network Open.

N202	Calculus of kidney with calculus of ureter
N209	Urinary calculus, unspecified
N210	Calculus in bladder
N211	Calculus in urethra
N218	Other lower urinary tract calculus
N219	Calculus of lower urinary tract, unspecified
N220	Urinary calculus in schistosomiasis [bilharziasis]
N228	Calculus of urinary tract in other diseases classified elsewhere
N23	Unspecified renal colic
Obstruction	
N130	Hydronephrosis with ureteropelvic junction obstruction
N131	Hydronephrosis with ureteral stricture, not elsewhere classified
N133	Other and unspecified hydronephrosis
N134	Hydroureter
N135	Kinking and stricture of ureter without hydronephrosis
N138	Other obstructive and reflux uropathy
N139	Obstructive and reflux uropathy, unspecified
N178	Other acute renal failure
N179	Acute renal failure, unspecified
N281	Cyst of kidney, acquired
N320	Bladder-neck obstruction
N350	Post-traumatic urethral stricture
N351	Postinfective urethral stricture, not elsewhere classified
N358	Other urethral stricture
N359	Urethral stricture, unspecified
**Penile and Urethral Disorders**	
N361	Urethral diverticulum
N362	Urethral caruncle
N363	Prolapsed urethral mucosa
N368	Other specified disorders of urethra
N369	Urethral disorder, unspecified
N378	Other urethral disorders in diseases classified elsewhere
N470	Phimosis
N471	Paraphimosis
N478	Other disorders of prepuce
N481	Balanoposthitis
N4820	Abscess of penis
N4821	Cellulitis of penis
N4828	Other inflammatory disorders of penis
N483	Priapism
N4840	Male erectile dysfunction due to arterial insufficiency
N4841	Male erectile dysfunction due to corporovenous occlusive dysfunction
N4848	Male erectile dysfunction due to other cause
N4849	Male erectile dysfunction, unspecified
N485	Ulcer of penis
N488	Other specified disorders of penis
N489	Disorder of penis, unspecified
N5018	Other vascular disorders of male genital organs
N508	Other specified disorders of male genital organs
N509	Disorder of male genital organs, unspecified
**Prostate disorders**	
N40	Hyperplasia of prostate
N410	Acute prostatitis

© 2026 Matta R et al. JAMA Network Open.

N411	Chronic prostatitis
N412	Abscess of prostate
N413	Prostatocystitis
N419	Inflammatory disease of prostate, unspecified
N420	Calculus of prostate
N421	Congestion and haemorrhage of prostate
N422	Atrophy of prostate
N423	Dysplasia of prostate
N4280	Prostatodynia syndrome
N4288	Other specified disorders of prostate
N429	Disorder of prostate, unspecified
N510	Disorders of prostate in diseases classified elsewhere
**Scrotal disorders**	
N432	Other hydrocele
N433	Hydrocele, unspecified
N434	Spermatocele
N4500	Epididymitis with abscess
N4501	Orchitis with abscess
N4502	Epididymo-orchitis with abscess
N4590	Epididymitis
N4591	Orchitis
N4592	Epididymo-orchitis
N460	Azoospermia
N461	Oligospermia
N468	Other male infertility
N469	Unspecified male infertility
N490	Inflammatory disorders of seminal vesicle
N491	Inflammatory disorders of spermatic cord, tunica vaginalis and vas deferens
N492	Inflammatory disorders of scrotum
N498	Inflammatory disorders of other specified male genital organs
N499	Inflammatory disorder of unspecified male genital organ
N511	Disorders of testis and epididymis in diseases classified elsewhere

© 2026 Matta R et al. JAMA Network Open.

eMethods

We linked the following databases: the Ontario Health Insurance Plan (OHIP) database, which tracks claims paid for physician billings, laboratories, and out-of-province providers[54]; the Canadian Institute for Health Information Discharge Abstract Database (CIHI-DAD)[55], which contains records for hospital admissions; the CIHI National Ambulatory Care Reporting System, which contains diagnosis and procedure information during emergency department visits; the Registered Persons Database (RPDB), which captures vital statistics for all individuals alive and eligible for OHIP during the accrual period[56]; Postal Code Conversion File (PCCF), which contains postal codes within a given cohort and determines other census geographic identifiers such as, dissemination/enumeration area, census division, longitude/latitude, urban/rural flag and neighbourhood income quintile; and the ICES MOMBABY database, which is an ICES-derived cohort that links the DAD inpatient admission records of delivering mothers and their newborns. From 2002 onward, this linkage is performed deterministically using a maternal-newborn chart matching number. Prior to 2002, mothers were linked to their children by matching on the institutions they were admitted to, their postal codes, and their admission/discharge dates.

© 2026 Matta R et al. JAMA Network Open.

eTable 2. Cohort Selection for all emergency department visits

Step	Criteria	Description	# Excluded	Total Cohort Remaining	%
1	Inclusion	Number of records in NACRS with valid IKN between 1 Jan 2007 and 31 Dec 2022	Before Exclusion	87,063,273	
2	Exclusion	Invalid birth date, death date or gender	49,111	87,014,162	99.94
3	Exclusion	Non-Ontario residents	70,470	86,943,692	99.92
4	Exclusion	Age <18 or >105 years	16,441,210	70,502,482	81.09
5	Exclusion	Not eligible for OHIP	1,501,216	69,001,266	97.87
6	Exclusion	Pregnant women	1,861,934	67,139,332	97.30
7	Exclusion	Admissions with injuries due to external causes	14,710,867	52,428,465	78.09
		**Study Cohort (number of observations)**		**52,428,465**	
8		Taking the first episode in each calendar year for each patient	22,967,184	29,461,281	56.19
		**Study Cohort (unique patients per calendar year)**		**29,461,281**	

© 2026 Matta R et al. JAMA Network Open.

eTable 3. Cohort Selection for all emergency department visits with a primary urologic diagnosis

Step	Criteria	Description	# Excluded	Total Cohort Remaining	%
1	Inclusion	Number of records in NACRS with main diagnosis of urologic disorders and valid IKN between Jan 1 2007 and Dec 31 2022	Before Exclusion	3,137,909	
2	Exclusion	Invalid birth date, death date or gender	1,593	3,136,316	99.95
3	Exclusion	Non-Ontario residents	2,035	3,134,281	99.94
4	Exclusion	Age <18 or >105 years	316,856	2,817,425	89.89
5	Exclusion	Not eligible for OHIP	54,709	2,762,716	98.06
6	Exclusion	Pregnant women	36,293	2,726,423	98.69
7	Exclusion	Admissions with injuries due to external causes	18,476	2,707,947	99.32
		**Study Cohort (number of observations)**		**2,707,947**	
8		Taking the first episode in each calendar year for each patient	515,734	2,192,213	80.95
		**Study Cohort (unique patients per calendar year)**		**2,192,213**	

© 2026 Matta R et al. JAMA Network Open.

eTable 4 Baseline characteristics of patients seen in the emergency department with a primary urological diagnosis for a new urological condition

		Calendar year																P Trend
		2007	2008	2009	2010	2011	2012	2013	2014	2015	2016	2017	2018	2019	2020	2021	2022	
**Variables**	n	N=92791	N=95958	N=97451	N=102651	N=105895	N=110862	N=111733	N=114784	N=116009	N=117042	N=116357	N=117483	N=117903	N=99577	N=108219	N=107641	
**Age in Years at ED Visit**	Mean (SD)	49.03 (20.61)	49.36 (20.73)	49.86 (20.82)	50.47 (20.97)	50.55 (21.08)	51.21 (21.26)	51.56 (21.27)	51.96 (21.34)	52.46 (21.29)	53.03 (21.29)	53.17 (21.28)	53.56 (21.24)	53.74 (21.25)	53.69 (21.05)	54.17 (21.25)	54.31 (21.32)	
	Median (Q1-Q3)	47 (32-65)	48 (32-65)	48 (32-66)	49 (33-67)	49 (32-67)	50 (33-68)	51 (33-68)	51 (34-69)	52 (34-69)	53 (35-70)	53 (35-70)	54 (35-70)	54 (36-71)	54 (36-70)	55 (36-71)	55 (36-72)	
**Sex on RPDB**	F	63739 (68.7)	66646 (69.5)	66957 (68.7)	70743 (68.9)	72928 (68.9)	76207 (68.7)	76764 (68.7)	78392 (68.3)	78365 (67.6)	78235 (66.8)	77083 (66.2)	77098 (65.6)	76208 (64.6)	60411 (60.7)	66257 (61.2)	65716 (61.1)	<0.0001
	M	29052 (31.3)	29312 (30.5)	30494 (31.3)	31908 (31.1)	32967 (31.1)	34655 (31.3)	34969 (31.3)	36392 (31.7)	37644 (32.4)	38807 (33.2)	39274 (33.8)	40385 (34.4)	41695 (35.4)	39166 (39.3)	41962 (38.8)	41925 (38.9)	
**Income Quintile**	1 - Lowest	20468 (22.1)	21402 (22.3)	21490 (22.1)	22305 (21.7)	22824 (21.6)	23763 (21.4)	23904 (21.4)	26834 (23.4)	26963 (23.2)	27414 (23.4)	27034 (23.2)	27189 (23.1)	27043 (22.9)	22841 (22.9)	24337 (22.5)	24641 (22.9)	<0.0001
	2	19047 (20.5)	19641 (20.5)	20161 (20.7)	21164 (20.6)	21860 (20.6)	22910 (20.7)	22644 (20.3)	24271 (21.1)	24712 (21.3)	24545 (21.0)	24191 (20.8)	24522 (20.9)	24647 (20.9)	20696 (20.8)	22549 (20.8)	22119 (20.5)	
	3	18491 (19.9)	18781 (19.6)	19073 (19.6)	20306 (19.8)	21066 (19.9)	22178 (20.0)	22635 (20.3)	22953 (20.0)	22978 (19.8)	23345 (19.9)	23335 (20.1)	23559 (20.1)	23746 (20.1)	19935 (20.0)	21823 (20.2)	21613 (20.1)	
	4	17859 (19.2)	18844 (19.6)	18975 (19.5)	20235 (19.7)	20820 (19.7)	21925 (19.8)	22166 (19.8)	20939 (18.2)	21413 (18.5)	21503 (18.4)	21678 (18.6)	22147 (18.9)	22168 (18.8)	18815 (18.9)	20515 (19.0)	20477 (19.0)	
	5 - Highest	16510 (17.8)	16777 (17.5)	17119 (17.6)	18054 (17.6)	18657 (17.6)	19405 (17.5)	19678 (17.6)	19360 (16.9)	19556 (16.9)	19759 (16.9)	19691 (16.9)	19668 (16.7)	19883 (16.9)	16927 (17.0)	18583 (17.2)	18368 (17.1)	
	Missing	416 (0.4)	513 (0.5)	633 (0.6)	587 (0.6)	668 (0.6)	681 (0.6)	706 (0.6)	427 (0.4)	387 (0.3)	476 (0.4)	428 (0.4)	398 (0.3)	416 (0.4)	363 (0.4)	412 (0.4)	423 (0.4)	
**Rurality**	Urban (community size >10,000 persons)	72349 (78.0)	74673 (77.8)	76164 (78.2)	80661 (78.6)	83841 (79.2)	88189 (79.5)	89131 (79.8)	92583 (80.7)	93830 (80.9)	94983 (81.2)	94463 (81.2)	95168 (81.0)	95604 (81.1)	80964 (81.3)	88083 (81.4)	88107 (81.9)	<0.0001
	Rural (community size ≤10,000 persons)	20409 (22.0)	21238 (22.1)	*21282-21286	*21985-21989	*22049-22053	22673 (20.5)	*22597-22601	21858 (19.0)	21847 (18.8)	21675 (18.5)	21532 (18.5)	21982 (18.7)	21945 (18.6)	18333 (18.4)	19796 (18.3)	19189 (17.8)	
	Missing	33 (0.0)	47 (0.0)	*1-5	*1-5	*1-5	0 (0.0)	*1-5	343 (0.3)	332 (0.3)	384 (0.3)	362 (0.3)	333 (0.3)	354 (0.3)	280 (0.3)	340 (0.3)	345 (0.3)	
**Income Quintile+ Rurality**	1 - Lowest	15932 (17.2)	16599 (17.3)	16724 (17.2)	17459 (17.0)	17956 (17.0)	18670 (16.8)	18955 (17.0)	21561 (18.8)	21839 (18.8)	22246 (19.0)	21890 (18.8)	21963 (18.7)	21757 (18.5)	18331 (18.4)	19587 (18.1)	19930 (18.5)	<0.0001
	2	14840 (16.0)	15283 (15.9)	15745 (16.2)	16598 (16.2)	17239 (16.3)	18140 (16.4)	17946 (16.1)	19648 (17.1)	20033 (17.3)	19865 (17.0)	19604 (16.8)	19900 (16.9)	20033 (17.0)	16848 (16.9)	18363 (17.0)	18138 (16.9)	
	3	14432 (15.6)	14651 (15.3)	14971 (15.4)	16045 (15.6)	16791 (15.9)	17701 (16.0)	18194 (16.3)	18577 (16.2)	18587 (16.0)	18938 (16.2)	18969 (16.3)	19165 (16.3)	19321 (16.4)	16321 (16.4)	17907 (16.5)	17815 (16.6)	
	4	14015 (15.1)	14747 (15.4)	14993 (15.4)	16112 (15.7)	16722 (15.8)	17831 (16.1)	17978 (16.1)	16935 (14.8)	17369 (15.0)	17599 (15.0)	17767 (15.3)	17990 (15.3)	18149 (15.4)	15449 (15.5)	16921 (15.6)	17047 (15.8)	
	5 - Highest	12930 (13.9)	13184 (13.7)	13470 (13.8)	14177 (13.8)	14807 (14.0)	15544 (14.0)	15718 (14.1)	15802 (13.8)	15964 (13.8)	16257 (13.9)	16192 (13.9)	16106 (13.7)	16307 (13.8)	13952 (14.0)	15257 (14.1)	15122 (14.0)	
	Rural (community size ≤10,000 persons)	20409 (22.0)	21238 (22.1)	21285 (21.8)	21986 (21.4)	22052 (20.8)	22673 (20.5)	22600 (20.2)	21858 (19.0)	21847 (18.8)	21675 (18.5)	21532 (18.5)	21982 (18.7)	21945 (18.6)	18333 (18.4)	19796 (18.3)	19189 (17.8)	
	Missing	233 (0.3)	256 (0.3)	263 (0.3)	274 (0.3)	328 (0.3)	303 (0.3)	342 (0.3)	403 (0.4)	370 (0.3)	462 (0.4)	403 (0.3)	377 (0.3)	391 (0.3)	343 (0.3)	388 (0.4)	400 (0.4)	
**ED Visit Discharge Disposition**	Discharged	85301 (91.9)	88039 (91.7)	88842 (91.2)	93318 (90.9)	95957 (90.6)	99710 (89.9)	100223 (89.7)	102621 (89.4)	103476 (89.2)	104046 (88.9)	103591 (89.0)	104224 (88.7)	104238 (88.4)	86763 (87.1)	94499 (87.3)	93791 (87.1)	<0.0001
	Admitted to hospital	7075 (7.6)	7474 (7.8)	8107 (8.3)	8791 (8.6)	9428 (8.9)	10545 (9.5)	10964 (9.8)	11599 (10.1)	11929 (10.3)	12352 (10.6)	12117 (10.4)	12591 (10.7)	12895 (10.9)	12141 (12.2)	12956 (12.0)	13053 (12.1)	
	Missing	415 (0.4)	445 (0.5)	502 (0.5)	542 (0.5)	510 (0.5)	607 (0.5)	546 (0.5)	564 (0.5)	604 (0.5)	644 (0.6)	649 (0.6)	668 (0.6)	770 (0.7)	673 (0.7)	764 (0.7)	797 (0.7)	
**Length of Hospital Stay in Days**	Mean (SD)	11.27 (27.7)	12.76 (75.5)	10.97 (20.54)	12.2 (62.21)	11.35 (42.03)	11.5 (75.99)	12.01 (96.55)	11.48 (103.72)	11.55 (81.3)	11.68 (65.13)	10.92 (46.9)	12.95 (155.19)	11.64 (53.19)	12.75 (64.45)	12.08 (28.72)	11.34 (17.5)	0.5706
	Median (Q1-Q3)	5 (3-11)	6 (3-12)	5 (3-12)	5 (3-11)	5 (3-11)	5 (3-11)	5 (3-10)	5 (3-10)	5 (3-10)	5 (3-10)	5 (3-10)	5 (3-11)	5 (3-11)	6 (3-11)	6 (3-12)	6 (3-12)	<0.0001
	None (0)	85716 (92.4)	88484 (92.2)	89344 (91.7)	93860 (91.4)	96468 (91.1)	100325 (90.5)	100777 (90.2)	103192 (89.9)	104084 (89.7)	104690 (89.4)	104240 (89.6)	104893 (89.3)	105008 (89.1)	87438 (87.8)	95264 (88.0)	94589 (87.9)	<0.0001
	1 to 2	1554 (1.7)	1528 (1.6)	1786 (1.8)	1970 (1.9)	2057 (1.9)	2422 (2.2)	2376 (2.1)	2556 (2.2)	2669 (2.3)	2839 (2.4)	2718 (2.3)	2787 (2.4)	2831 (2.4)	2375 (2.4)	2446 (2.3)	2593 (2.4)	
	3 to 7	2889 (3.1)	3034 (3.2)	3223 (3.3)	3538 (3.4)	3799 (3.6)	4343 (3.9)	4574 (4.1)	4916 (4.3)	5001 (4.3)	5223 (4.5)	5084 (4.4)	5229 (4.5)	5362 (4.5)	5046 (5.1)	5250 (4.9)	5114 (4.8)	

© 2026 Matta R et al. JAMA Network Open.

	>+8	2632 (2.8)	2912 (3.0)	3098 (3.2)	3283 (3.2)	3571 (3.4)	3772 (3.4)	4006 (3.6)	4120 (3.6)	4255 (3.7)	4290 (3.7)	4315 (3.7)	4574 (3.9)	4702 (4.0)	4718 (4.7)	5259 (4.9)	5345 (5.0)	
**ICU Admission**	Yes	690 (9.8)	759 (10.2)	795 (9.8)	923 (10.5)	912 (9.7)	1000 (9.5)	1138 (10.4)	1191 (10.3)	1247 (10.5)	1219 (9.9)	1145 (9.4)	1138 (9.0)	1168 (9.1)	1164 (9.6)	1211 (9.3)	1085 (8.3)	<0.0001
	No	6385 (90.2)	6715 (89.8)	7312 (90.2)	7868 (89.5)	8516 (90.3)	9545 (90.5)	9826 (89.6)	10408 (89.7)	10682 (89.5)	11133 (90.1)	10972 (90.6)	11453 (91.0)	11727 (90.9)	10977 (90.4)	11745 (90.7)	11968 (91.7)	
	Not applicable	85716	88484	89344	93860	96467	100317	100769	103185	104080	104690	104240	104892	105008	87436	95263	94588	
**In Hospital Mortality**	Yes	724 (10.2)	809 (10.8)	837 (10.3)	879 (10.0)	887 (9.4)	981 (9.3)	932 (8.5)	1004 (8.7)	1033 (8.7)	1037 (8.4)	987 (8.1)	1089 (8.6)	1135 (8.8)	1157 (9.5)	1140 (8.8)	1295 (9.9)	<0.0001
	No	6351 (89.8)	6665 (89.2)	7270 (89.7)	7912 (90.0)	8541 (90.6)	9564 (90.7)	10032 (91.5)	10595 (91.3)	10896 (91.3)	11315 (91.6)	11130 (91.9)	11502 (91.4)	11760 (91.2)	10984 (90.5)	11816 (91.2)	11758 (90.1)	
	Not applicable	85716	88484	89344	93860	96467	100317	100769	103185	104080	104690	104240	104892	105008	87436	95263	94588	
**Visit to Outpatient Urology Specialist**	Yes	9996 (11)	10335 (11)	10872 (11.4)	11671 (11.6)	11809 (11.4)	12409 (11.4)	13269 (12.1)	13504 (12)	14282 (12.5)	14636 (12.7)	14713 (12.9)	15417 (13.4)	15516 (13.4)	15482 (15.9)	17177 (16.2)	16116 (15.3)	<0.0001
	No	81107 (89)	84044 (89)	84841 (88.6)	89294 (88.4)	92233 (88.6)	96302 (88.6)	96552 (87.9)	99244 (88)	99718 (87.5)	100344 (87.3)	99589 (87.1)	99986 (86.6)	100321 (86.6)	81980 (84.1)	88722 (83.8)	89199 (84.7)	
	Not applicable	1688	1579	1738	1686	1853	2151	1912	2036	2009	2062	2055	2080	2066	2115	2320	2326	
**Wait Time in Days for Urology Specialist**	Mean (SD)	64.87 (79.95)	66.27 (80.62)	64.51 (79.42)	63.51 (79.14)	63.46 (78.86)	71.51 (89.92)	85.78 (88.93)	87.58 (89.03)	85.64 (87.85)	84.97 (87.21)	84.30 (86.24)	83.85 (85.69)	82.16 (84.90)	83.98 (87.52)	80.08 (82.45)	82.59 (80.86)	<0.0001
	Median (Q1-Q3)	29 (8-90)	31 (9-95)	29 (8-90)	29 (8-87)	28 (9-87)	31 (9-98)	51 (20-123)	53 (21-126)	52 (20-122)	52 (20-120)	51 (21-120)	52 (20-120)	51 (21-112)	48 (20-121)	49 (22-109)	53 (23-116)	<0.0001
**Number of ED Visits Until Urology Specialist**	Mean (SD)	1.19 (0.51)	1.21 (0.55)	1.21 (0.53)	1.23 (0.56)	1.23 (0.56)	1.25 (0.59)	1.29 (0.63)	1.29 (0.63)	1.29 (0.64)	1.3 (0.66)	1.29 (0.63)	1.31 (0.69)	1.31 (0.66)	1.31 (0.67)	1.31 (0.66)	1.28 (0.63)	<0.0001
	Median (Q1-Q3)	1 (1-1)	1 (1-1)	1 (1-1)	1 (1-1)	1 (1-1)	1 (1-1)	1 (1-1)	1 (1-1)	1 (1-1)	1 (1-1)	1 (1-1)	1 (1-1)	1 (1-1)	1 (1-1)	1 (1-1)	1 (1-1)	
**Continuity of Care**	Very low	42377 (45.7)	42779 (44.6)	42883 (44.0)	44694 (43.5)	46575 (44.0)	48376 (43.6)	50112 (44.8)	53069 (46.2)	53941 (46.5)	54579 (46.6)	55314 (47.5)	56756 (48.3)	57871 (49.1)	53872 (54.1)	72177 (66.7)	65483 (60.8)	<0.0001
	Low	9822 (10.6)	10152 (10.6)	10398 (10.7)	11029 (10.7)	11463 (10.8)	12054 (10.9)	11923 (10.7)	12109 (10.5)	12356 (10.7)	12795 (10.9)	12549 (10.8)	12984 (11.1)	12878 (10.9)	9385 (9.4)	5888 (5.4)	7598 (7.1)	
	Medium	8300 (8.9)	8324 (8.7)	8254 (8.5)	8762 (8.5)	8634 (8.2)	8871 (8.0)	8459 (7.6)	8467 (7.4)	8578 (7.4)	8774 (7.5)	8540 (7.3)	8490 (7.2)	8423 (7.1)	4983 (5.0)	1800 (1.7)	2999 (2.8)	
	High	31258 (33.7)	33621 (35.0)	34822 (35.7)	36798 (35.8)	37689 (35.6)	39929 (36.0)	39567 (35.4)	39333 (34.3)	39146 (33.7)	38794 (33.1)	38003 (32.7)	37235 (31.7)	36564 (31.0)	29227 (29.4)	25740 (23.8)	28607 (26.6)	
	No UPC identified	1034 (1.1)	1082 (1.1)	1094 (1.1)	1368 (1.3)	1534 (1.4)	1632 (1.5)	1672 (1.5)	1806 (1.6)	1988 (1.7)	2100 (1.8)	1951 (1.7)	2018 (1.7)	2167 (1.8)	2110 (2.1)	2614 (2.4)	2954 (2.7)	
**Physician visit within 6 months of ED visit**	No	44957 (48.4)	46249 (48.2)	46582 (47.8)	48744 (47.5)	51086 (48.2)	53952 (48.7)	55800 (49.9)	58378 (50.9)	59180 (51.0)	59709 (51.0)	60520 (52.0)	61740 (52.6)	62939 (53.4)	67127 (67.4)	80205 (74.1)	71684 (66.6)	<0.0001
	Yes	46800 (50.4)	48627 (50.7)	49775 (51.1)	52539 (51.2)	53275 (50.3)	55278 (49.9)	54261 (48.6)	54600 (47.6)	54841 (47.3)	55233 (47.2)	53886 (46.3)	53725 (45.7)	52797 (44.8)	30340 (30.5)	25400 (23.5)	33003 (30.7)	
	No UPC identified	1034 (1.1)	1082 (1.1)	1094 (1.1)	1368 (1.3)	1534 (1.4)	1632 (1.5)	1672 (1.5)	1806 (1.6)	1988 (1.7)	2100 (1.8)	1951 (1.7)	2018 (1.7)	2167 (1.8)	2110 (2.1)	2614 (2.4)	2954 (2.7)	
**Charlson Comorbidity Index**	0	75687 (81.6)	77213 (80.5)	77511 (79.5)	80870 (78.8)	83254 (78.6)	86090 (77.7)	86076 (77.0)	88175 (76.8)	88327 (76.1)	88589 (75.7)	88191 (75.8)	88715 (75.5)	89210 (75.7)	75387 (75.7)	82735 (76.5)	82196 (76.4)	<0.0001
	1	9589 (10.3)	10309 (10.7)	10757 (11.0)	11737 (11.4)	12131 (11.5)	13078 (11.8)	13365 (12.0)	13626 (11.9)	13885 (12.0)	14070 (12.0)	14012 (12.0)	14235 (12.1)	14046 (11.9)	11563 (11.6)	11738 (10.8)	11722 (10.9)	
	2	3603 (3.9)	3907 (4.1)	4187 (4.3)	4804 (4.7)	4982 (4.7)	5642 (5.1)	5951 (5.3)	6238 (5.4)	6649 (5.7)	6901 (5.9)	6800 (5.8)	7057 (6.0)	7111 (6.0)	6151 (6.2)	6760 (6.2)	6825 (6.3)	
	3+	3912 (4.2)	4529 (4.7)	4996 (5.1)	5240 (5.1)	5528 (5.2)	6052 (5.5)	6341 (5.7)	6745 (5.9)	7148 (6.2)	7482 (6.4)	7354 (6.3)	7476 (6.4)	7536 (6.4)	6476 (6.5)	6986 (6.5)	6898 (6.4)	

Define abbreviations - ICU ; SD ; Q1 ; Q3 ; UPC ; ED

© 2026 Matta R et al. JAMA Network Open.

eTable 5 Age- and sex- standardized rates of all unique ED visits and a first ED visit with a primary urological diagnosis for new urologic conditions between Jan 1 2007 and Dec 31 2022 by year

	AGE- (AND SEX-) STANDARDIZED RATES PER 100											
	First ED visit for new urologic disorders (unique by year)						All unique ED visits (unique by year)					
Calendar year	Females		Males		Total		Females		Males		Total	
	Rate (95% CI)	RR (95% CI)	Rate (95% CI)	RR (95% CI)	Rate (95% CI)	RR (95% CI)	Rate (95% CI)	RR (95% CI)	Rate (95% CI)	RR (95% CI)	Rate (95% CI)	RR (95% CI)
2007	1.2 (1.19-1.21)	Ref.	0.6 (0.59-0.6)	Ref.	0.91 (0.9-0.91)	Ref.	16.17 (16.14-16.21)	Ref.	14.87 (14.83-14.9)	Ref.	15.5 (15.47-15.52)	Ref.
2008	1.24 (1.23-1.25)	1.03 (1.02-1.05)	0.6 (0.59-0.6)	1 (0.98-1.01)	0.93 (0.92-0.93)	1.02 (1.01-1.03)	16.32 (16.29-16.36)	1.01 (1.01-1.01)	14.9 (14.86-14.93)	1 (1-1.01)	15.59 (15.57-15.61)	1.01 (1-1.01)
2009	1.23 (1.22-1.24)	1.02 (1.01-1.03)	0.61 (0.6-0.61)	1.02 (1-1.03)	0.92 (0.92-0.93)	1.02 (1.01-1.03)	16.61 (16.57-16.64)	1.03 (1.02-1.03)	14.92 (14.89-14.95)	1 (1-1.01)	15.75 (15.72-15.77)	1.02 (1.01-1.02)
2010	1.27 (1.26-1.28)	1.06 (1.05-1.07)	0.62 (0.62-0.63)	1.04 (1.03-1.06)	0.96 (0.95-0.96)	1.05 (1.04-1.06)	16.6 (16.56-16.63)	1.03 (1.02-1.03)	14.89 (14.85-14.92)	1 (1-1)	15.73 (15.7-15.75)	1.01 (1.01-1.02)
2011	1.29 (1.28-1.3)	1.07 (1.06-1.08)	0.63 (0.62-0.64)	1.06 (1.04-1.07)	0.97 (0.96-0.97)	1.07 (1.06-1.08)	16.93 (16.89-16.96)	1.05 (1.04-1.05)	15.13 (15.1-15.17)	1.02 (1.02-1.02)	16.02 (15.99-16.04)	1.03 (1.03-1.04)
2012	1.32 (1.31-1.33)	1.1 (1.09-1.11)	0.65 (0.64-0.66)	1.09 (1.07-1.1)	0.99 (0.99-1)	1.1 (1.09-1.11)	17.4 (17.37-17.44)	1.08 (1.07-1.08)	15.43 (15.4-15.47)	1.04 (1.04-1.04)	16.41 (16.38-16.43)	1.06 (1.06-1.06)
2013	1.3 (1.3-1.31)	1.09 (1.08-1.1)	0.64 (0.64-0.65)	1.08 (1.06-1.09)	0.98 (0.98-0.99)	1.09 (1.08-1.09)	17.19 (17.15-17.22)	1.06 (1.06-1.07)	15.2 (15.17-15.24)	1.02 (1.02-1.03)	16.18 (16.16-16.21)	1.04 (1.04-1.05)
2014	1.32 (1.31-1.33)	1.1 (1.09-1.11)	0.66 (0.66-0.67)	1.11 (1.09-1.13)	1 (1-1.01)	1.1 (1.09-1.11)	17.54 (17.5-17.57)	1.08 (1.08-1.09)	15.53 (15.49-15.56)	1.04 (1.04-1.05)	16.51 (16.49-16.54)	1.07 (1.06-1.07)
2015	1.3 (1.29-1.31)	1.09 (1.08-1.1)	0.68 (0.67-0.68)	1.13 (1.11-1.15)	1 (1-1.01)	1.1 (1.09-1.11)	17.59 (17.56-17.62)	1.09 (1.09-1.09)	15.59 (15.56-15.63)	1.05 (1.04-1.05)	16.57 (16.55-16.59)	1.07 (1.07-1.07)
2016	1.28 (1.27-1.29)	1.07 (1.06-1.08)	0.69 (0.68-0.69)	1.14 (1.12-1.16)	0.99 (0.99-1)	1.09 (1.08-1.1)	17.75 (17.72-17.78)	1.1 (1.09-1.1)	15.79 (15.76-15.82)	1.06 (1.06-1.06)	16.75 (16.73-16.77)	1.08 (1.08-1.08)
2017	1.24 (1.23-1.25)	1.04 (1.03-1.05)	0.68 (0.67-0.69)	1.13 (1.12-1.15)	0.97 (0.96-0.98)	1.07 (1.06-1.08)	17.76 (17.73-17.79)	1.1 (1.1-1.1)	15.76 (15.73-15.79)	1.06 (1.06-1.06)	16.74 (16.72-16.77)	1.08 (1.08-1.08)
2018	1.22 (1.21-1.23)	1.02 (1.01-1.03)	0.69 (0.68-0.69)	1.14 (1.13-1.16)	0.96 (0.95-0.97)	1.06 (1.05-1.07)	17.67 (17.64-17.71)	1.09 (1.09-1.1)	15.65 (15.62-15.68)	1.05 (1.05-1.05)	16.65 (16.62-16.67)	1.07 (1.07-1.08)
2019	1.18 (1.17-1.19)	0.99 (0.98-1)	0.69 (0.69-0.7)	1.15 (1.14-1.17)	0.94 (0.94-0.95)	1.04 (1.03-1.05)	17.37 (17.33-17.4)	1.07 (1.07-1.08)	15.37 (15.34-15.4)	1.03 (1.03-1.04)	16.35 (16.33-16.38)	1.06 (1.05-1.06)
2020	0.93 (0.92-0.94)	0.78 (0.77-0.79)	0.65 (0.64-0.65)	1.08 (1.07-1.1)	0.8 (0.79-0.8)	0.88 (0.87-0.88)	15.06 (15.03-15.09)	0.93 (0.93-0.93)	13.93 (13.9-13.96)	0.94 (0.93-0.94)	14.47 (14.45-14.49)	0.93 (0.93-0.94)
2021	1 (0.99-1.01)	0.84 (0.83-0.85)	0.68 (0.67-0.69)	1.14 (1.12-1.15)	0.85 (0.84-0.85)	0.93 (0.92-0.94)	15.73 (15.7-15.76)	0.97 (0.97-0.98)	14.28 (14.25-14.31)	0.96 (0.96-0.96)	14.98 (14.96-15.01)	0.97 (0.96-0.97)
2022	0.97 (0.96-0.98)	0.81 (0.8-0.82)	0.66 (0.66-0.67)	1.11 (1.09-1.12)	0.82 (0.82-0.83)	0.91 (0.9-0.91)	15.66 (15.63-15.69)	0.97 (0.97-0.97)	13.9 (13.87-13.93)	0.93 (0.93-0.94)	14.76 (14.74-14.78)	0.95 (0.95-0.95)
**Total**	**1.2 (1.2-1.2)**		**0.65 (0.65-0.66)**		**0.94 (0.94-0.94)**		**16.82 (16.81-16.83)**		**15.05 (15.05-15.06)**		**15.92 (15.91-15.92)**	

This table shows the crude and age- and sex-standardized rates of first ED visit with a primary urological diagnosis for new urologic conditions (excluding those with a urologic related visit in the previous 2 years, and then taking the first episode in each calendar year for each patient) by calendar year (2007 to 2022) for females, males and the total sample. See Figure 1. Denominator = Ontario residents eligible for OHIP each year, Standard population = 2014 Ontario population eligible for OHIP with 5 year age groups (18-19, 20-24, ..., 85-89, 90+). Rate ratios and 95% CI were estimated using the EFFECT option (PROC STDRATE in SAS), which computes the rate effect between the study populations (years, 2007 as the reference) with the default rate ratio statistics. For example, the standardized rate of unique ED visits with main diagnosis of urologic disorders for new urologic conditions among females in 2008 was 3% more than that in 2007 (RR 1.03, 95% CI 1.02-1.05).

© 2026 Matta R et al. JAMA Network Open.

eTable 6 Association between patient characteristics and hospital admission and wait time to see urologist as an outpatient following emergency department (ED) visits for new urological diagnoses, crude model

	Hospital admission	Wait time to see urologist
	**Odds Ratios (95% CI)**	**Hazard Ratios (95% CI)**
**Age**	1.07 (1.07-1.07)	1.01 (1.01 - 1.01)
**Sex**		
Male	1.00 (Ref.)	1.00 (Ref.)
Female	0.75 (0.75 - 0.76)	0.29 (0.29 - 0.29)
**Income Quintile**		
5 - highest	1.00 (Ref.)	1.00 (Ref.)
4	1 (0.98 - 1.02)	0.97 (0.96 – 0.99)
3	1.1 (1.08 - 1.12)	0.91 (0.9 - 0.92)
2	1.24 (1.22 - 1.27)	0.88 (0.86 - 0.95)
1 - lowest	1.4 (1.37 - 1.42)	0.79 (0.78 - 0.8)
**Charlson Comorbidity Index**		
0	1.00 (Ref.)	1.00 (Ref.)
1	3.19 (3.14 - 3.23)	1.00 (0.99 - 1.01)
2	11.23 (11.05 - 11.41)	0.84 (0.83 - 0.86)
3+	17.28 (17.02 - 17.55)	0.64 (0.63 - 0.66)
**Continuity of Care**[c]		
Very low	1.00 (Ref.)	1.00 (Ref.)
None	0.97 (0.93 - 1.01)	1.09 (1.05 - 1.13)
Low	0.89 (0.88 - 0.91)	1.1 (1.08 - 1.11)
Medium	1.24 (1.21 - 1.26)	1.2 (1.18 - 1.22)
High	1.13 (1.12 - 1.14)	1.15 (1.14 - 1.17)

[a] Patient data from first ED visit in study period
[b] n = 1,548,641
[c] Bice-Boxerman Continuity of Care Index
CI – confidence interval

© 2026 Matta R et al. JAMA Network Open.
