# Supplementary material for: Emergency Department Visits, Hospital Admissions, and Wait Times for Patients With Urologic Conditions
Source: JAMA Netw Open. 2026 Mar 9;9(3):e2560058. doi: 10.1001/jamanetworkopen.2025.60058 (PMC12973099; doi:10.1001/jamanetworkopen.2025.60058)
Supplement: Supplement 2. — Data Sharing Statement [file jamanetwopen-e2560058-s002.pdf]

Data Sharing Statement

Matta. Emergency Department Visits, Hospital Admissions, and Wait Times for Patients With Urologic Conditions. *JAMA Netw Open*. Published February 27, 2026.
doi:10.1001/jamanetworkopen.2025.60058

Data
Data available: No

Additional Information
Explanation for why data not available: This data is housed at ICES and is available to researchers at public institutions upon request.
